# Facile preparation of a novel nickel-containing metallopolymer via RAFT polymerization

**DOI:** 10.1080/15685551.2016.1257378

**Published:** 2016-11-21

**Authors:** Rong Ren, Yanhua Wang, Dizheng Liu, Weilin Sun

**Affiliations:** ^a^ MOE Key Laboratory of Macromolecular Synthesis and Functionalization, Department of Polymer Science and Engineering, Zhejiang University, Hangzhou, China

**Keywords:** Metallopolymer, RAFT polymerization

## Abstract

While the metallocene polymers were comprehensively studied, other metallopolymers are rarely explored. The major challenge is the lack of a synthetic platform for the preparation of metal coordinated derivatives, monomers, and polymers. Therefore, the development of a facile synthesis of new metal coordinated monomers and polymers is critically needed. A novel successfully synthesized methacrylate-containing nickel complex is reported in this communication. Controlled RAFT polymerizations are further carried out to prepare a series of side-chain nickel containing polymers with different molecular weight and narrow Polydispersity Index (PDI). This new metallopolymer performs specific electrochemical and excellent thermal properties. This study provides a novel and convenient strategy to prepare metallopolymer with controllable molecular weight, which has potential applications in assembled, catalytic and magnetic materials.

## 1. Introduction

Metallopolymers have attracted great attention recently because they have high synthetic efficiencies, readily processabilities and incredible versatilities as organic polymer frames complexed with inorganic metals to have specific redox, responsive, magnetic and catalytic properties.[1–10] Therefore, metallopolymer was described as a Pandora’s box of new materials by Manners.[11] With the introduction of metal centers into polymer matrix, a Pandora’s Box of fascinating new materials with potentially useful properties has been opened.

Metallocene is well known for its good chemical stability, redox responsibility and magnet property, therefore, studies on metallopolymer have been mostly focused on synthesizing metallocene-containing polymers.[12–24] Main-chain and side-chain metallocene-containing polymers can be prepared by ring opening polymerization (ROP), controlled radical polymerization and ring-opening metathesis polymerization (ROMP), respectively.[12,18,21,25,26]

Compared with metallocene polymers, other homopolymerized metallopolymers are rarely explored. The major challenge is the lack of a synthetic platform for the preparation of metal coordinated derivatives, monomers and polymers. Bin Chen synthesized a copolymer with pendant lanthanide complexes by using high yielding Diels–Alder cycloaddition to ‘click’ lanthanide complexes into polymer matrix.[27] Moreover, complicated polypentafluorophenyl acrylate copolymers were synthesized by Yang Li through reversible addition-fragmentation chain transfer (RAFT) polymerization, and were used as scaffolds to attach Gd^3+^ chelates for preparing gadolinium contrast agents via a ‘grafting to’ strategy.[28] Other ligands such as carbonyl, pyridine, hydroxyquinoline, porphyrin and diketone were also occasionally reported.[29–33] Nevertheless, most of the metallopolymers were copolymerization or post-polymerization modifications rather than direct polymerization of metal containing monomers, which lead to the random metallopolymer structure and less metal content. Therefore, the development of a facile synthesis of new metal coordinated monomers and polymers is critically needed.

Herein, we report a novel and convenient strategy to prepare a nickel metallopolymer with well-controlled molecular weight. We successfully synthesized a methacrylate-containing nickel complex HSNi and utilized it to prepare side-chain nickel containing polymers by using controlled RAFT polymerization. The structure and of the monomer and polymers were characterized by using ^1^H-NMR spectroscopy, GPC and Maldi-ToF. Furthermore, the thermal and redox properties were also investigated via DSC, TGA, and cyclic voltammetry. More fascinating properties such as assembled, catalytic and magnetic properties were waiting to be discovered in the Pandora’s box.

## 2. Experimental section

### 2.1. Materials and measurements

#### 2.1.1. Materials

The synthesis of cumyl dithiobenzoate (CDB) was conducted according to the literature procedure.[34] 1-Bromohexane, hydroxyethyl methylacrylate (HEMA), 2,4-dihydroxybenzaldehyde, tetrabutylammonium hexafluorophosphate (TBAPF_6_) and 3,4-diaminobenzoic acid, (≥98%) were purchased from J&K Chemical (China) and used as received. Tetrahydrofuran (THF) was refluxed over potassium/benzophenone until the solution turned blue and distilled before use. 2,2′-Azobis(2-methylpropionitrile) (AIBN, 99%) was recrystallized twice from ethanol and dried under vacuum at room temperature. Di-tert-butyl dicarbonate, 1-ethyl-3-(3-dimethylaminopropyl)carbodiimide hydrochloride (EDC·HCl), trifluoroacetic acid, K_2_CO_3_, KI, Ni(CH_3_COO)_2_·4H_2_O, Cu(CH_3_COO)_2_·4H_2_O, Zn(CH_3_COO)_2_·4H_2_O, and triethylamine were purchased from Sinopharm Chemical Reagent limited corporation (China) and used as received.

#### 2.1.2. Measurements

^1^H-NMR spectra in solution were obtained from 400 MHz Bruker DRX400 (Germany). Elemental analyses (EA) were carried out with an Elementar Vario MICRO (Germany). The valence of nickel was analysed by X-ray photoelectron spectroscopy (XPS) in a VG ESCALAB Mark II instrument (UK) using Mg-Kα excitation source. Gel-permeation chromatography (GPC) measurements were performed at 25 °C on a Waters 515 (US) equipped with Wyatt Technology Optilab rEX differential refractive index and UV detectors. THF was used as the eluent at a flow rate of 1.0 mL·min^−1^, the solvent THF and sample solutions were filtered over a filter with pore size of 0.45 μm (Nylon, Millex-HN 13 mm Syringes Filters, Millipore, US). The weight average (*M*_w_) and number average (*M*_n_) molecular weights were calculated with a calibration curve based on a group of polystyrene (PSt) standard samples. Matrix-assisted laser desorption/ionization time-of-flight mass spectrometry (MALDI-ToF-MS) was performed on an Autoflex II instrument (Bruker, Germany) and operating in linear mode. The analytical sample solution was prepared by mixing the solution of 1.0 mg sample in 100 μL THF with α-cyano-4-hydroxycinnamic acid (CHCA) saturated 10 μL THF solution. Then 1 μL of the mixed solution was deposited on the sample holder and allowed to dry at room temperature before inserted into the vacuum chamber of the MALDI-ToF instrument. A BAS CV-50 W voltammetric analyser (Japan) was used to perform cyclic voltammetry (CV) characterization. The electrode system included platinum disk working electrode, Pt counter-electrode and saturated calomel reference-electrode. A 1 mM solution in DCM of the compound was measured with 0.1 M TBAPF_6_ as the supporting electrolyte. Differential scanning calorimetry (DSC) thermograms were recorded on a Perkin-Elmer Pyris I calorimeter (US) equipped with a cooling accessory and under argon atmosphere. Typically, about 8 mg of the solid sample was encapsulated in a sealed aluminum pan with an identical empty pan as the reference. Indium was used as a calibration standard.

### 2.2. Synthesis of 4-hexyloxysalicylaldehyde

4-hexyloxysalicylaldehyde was prepared based on the general method given in the literature with some modifications.[35] 2,4-Dihydroxybenzaldehyde (10 mmol, 1.38 g), K_2_CO_3_ (10 mmol, 1.0 g), KI (catalytic amount) and 1-bromohexane (10 mmol, 1.65 g) were mixed in 25 mL of dry DMF. The mixture was heated under 80 °C for 15 h and then poured into water. Diluted HCl was added to neutralize the warm solution, which was then extracted with petroleum ether twice. After evaporated the petroleum ether, NaOH ethanol solution was added and heated at 80 °C until no solid residue left and cooled to room temperature. Then the white sodium phenolate was filtered and acidized by the mixture of diluted HCl and petroleum. At last, a white solid product was separated after the final evaporation of petroleum. Yield: 860 mg, 39%. ^1^H-NMR and ^13^C NMR (Figure S2, Supporting Information).

### 2.3. Synthesis of 3,4-di(tert-butoxycarbonylamino)benzoic acid (DBBA)

Di-tert-butyl dicarbonate (17.0 mL, 100 mmol) in 150 mL of dioxane was dropped in 150 mL aqueous solution of 3,4-diaminobenzoic acid (7.60 g, 50 mmol) and triethylamine (27.8 mL, 200 mmol) at room temperature. The reaction mixture was stirred overnight and then reduced approximately 50% of the original volume in vacuo. The remained solution was washed with diethyl ether and then extracted with ethyl acetate. After the mixture was cooled in an ice-water bath, the pH of the aqueous layer was adjusted to 2.5 via adding 1 mol/L of cold HCl. The ethyl acetate layer was then separated and washed with brine followed by drying over anhydrous sodium sulfate. The final product of DBBA was obtained as a gray solid after evaporation in vacuo and trituration with diethyl ether. Yield: 16.5 g, 94%. ^1^H-NMR (DMSO-d_6_, 400 M, *δ*, ppm): 12.81 (s, 1H, COOH), 8.77 (s, 1H, NH), 8.72 (s, 1H, NH), 8.09 (s, 1H, ArH), 7.68 (d, *J* = 24.1 Hz, 2H, ArH), 1.48 (s, 18H, CH_3_).

### 2.4. Synthesis of HEMA-DBBA

A mixture of DBBA (6.6 g, 32 mmol) and HEMA (4.2 g, 32 mmol) in CH_2_Cl_2_ (100 mL) was stirred at 0 °C. DMAP (1.9 g, 16 mmol) and EDC·HCl (9.2 g, 48 mmol) were added and the mixture was stirred overnight at room temperature. After washing with 1 M of HCl (100 mL) and NaHCO_3_ (100 mL) three times as well as brine once, the organic layer was dried over MgSO_4_. The solvent was removed by rotary evaporation, and then the product was further dried under vacuum. HEMA-DBBA was obtained as yellow oil. Yield: 10 g, 98%. ^1^H-NMR (CDCl_3_, 400 M, δ, ppm): 7.97 (s, 1H, ArH), 7.85 (m, 2H, ArH), 7.19 (s, 1H, NH), 6.51 (s, 1H, NH), 6.14, 5.58 (m, 2H, C=CH_2_), 4.51 (m, 4H, CH_2_CH_2_), 1.94 (s, 3H, CH_3_), 1.52 (s, 18H, CH_3_).

### 2.5. Synthesis of HEMA-DABA

HEMA-DBBA (0.223 g, 0.50 mmol) was dissolved in trifluoroacetic acid (5 mL) and stirred at room temperature for 5 h. The solvent was evaporated under reduced pressure, and the residue was triturated with saturated solution of Na_2_CO_3_ to obtain HEMA-DABA as a white solid. Yield: 130 mg, 98%. ^1^H-NMR (Figure S3).

### 2.6. Synthesis of HEMA-Salphen-M (HSM)

The synthesis of HEMA-Salphen-M complexes were prepared by a template method.[36] HEMA-DABA (0.500 mmol) was added to a solution of 4-hexyloxysalicylaldehyde (1.00 mmol) and M(CH_3_COO)_2_ (0.5 mmol, M = Ni, Cu, Zn) in ethanol (20 mL), under stirring. The mixture was heated at reflux with stirring for 4 h under nitrogen atmosphere. The precipitated product was cooled down and collected by filtration washing with ethanol. The HSNi could also be synthesized by esterification of the nickel-containing monomer precursor nickel-salphen and HEMA (Scheme S1). We prepared a universal diamine as a precursor to produce diverse metal salphen complexes with different metal salts. Yield: HSNi, 90%; HSCu, 84%; HSZn, 96%. ^1^H-NMR (Figure S5 and S6). Elemental analysis calculated (anal. calcd. %) for C_39_H_46_N_2_NiO_8_ (729.5): C 64.21, H 6.36, N 3.84; Found, C 63.05, H 6.57, N 4.02.

### 2.7. RAFT polymerization of HEMA-Salphen-Ni (HSNi)

A glass ampule was loaded with HSNi (365 mg, 0.5 mmol), AIBN (0.49 mg, 0.003 mmol), CDB (2.72 mg, 0.01 mmol) and THF (1.0 mL). The mixture was degassed through three freeze-pump-thaw cycles. The ampoule was then flame sealed under vacuum, and immersed into an oil bath thermo stated at 65 °C to start the polymerization. After 8 h, the ampule was quenched in liquid nitrogen to stop the polymerization. The reaction mixture was diluted with THF and precipitated in an excess of ethyl acetate. The purification was repeated for three times. The obtained product was dried overnight in a vacuum oven at room temperature. Anal. calcd. (%) for PHSNi: C 64.21, H 6.36, N 3.84; Found, C 63.08, H 6.58, N 4.01.

## 3. Results and discussion

### 3.1. Monomer synthesis

**Scheme 1.**
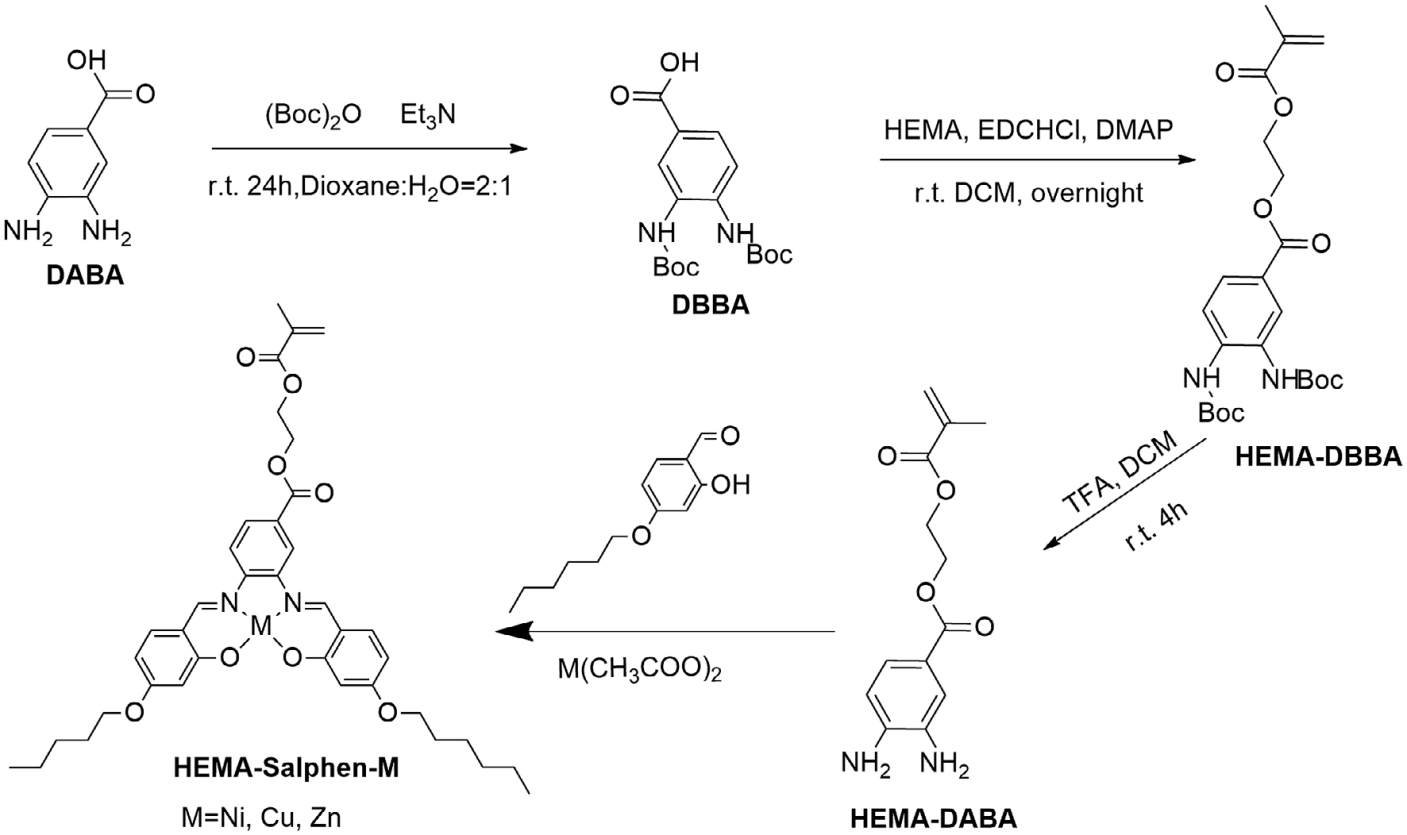
Synthetic procedure of HEMA-Salphen-M.

Scheme 1 shows the synthetic procedure of HSNi and its polymer PHSNi prepared by RAFT polymerization. HEMA-DBBA was synthesized by condensation of the HEMA and 3,4-diaminobenzoic acid protected by t-butyloxycarbonyl, in the presence of activating and condensating agents typically used for condensation reactions. Particularly, in order to avoid the formation of benzimidazole derivatives (Figure S3),[37] HSNi was obtained in a template synthesis using the Ni(CH_3_COO)_2_·4H_2_O, which prefers tetrahedral coordination with salicylaldehyde derivatives.[38] Monomers and polymers containing copper and zinc were accordingly systhesized in supporting information.

**Figure 1.**
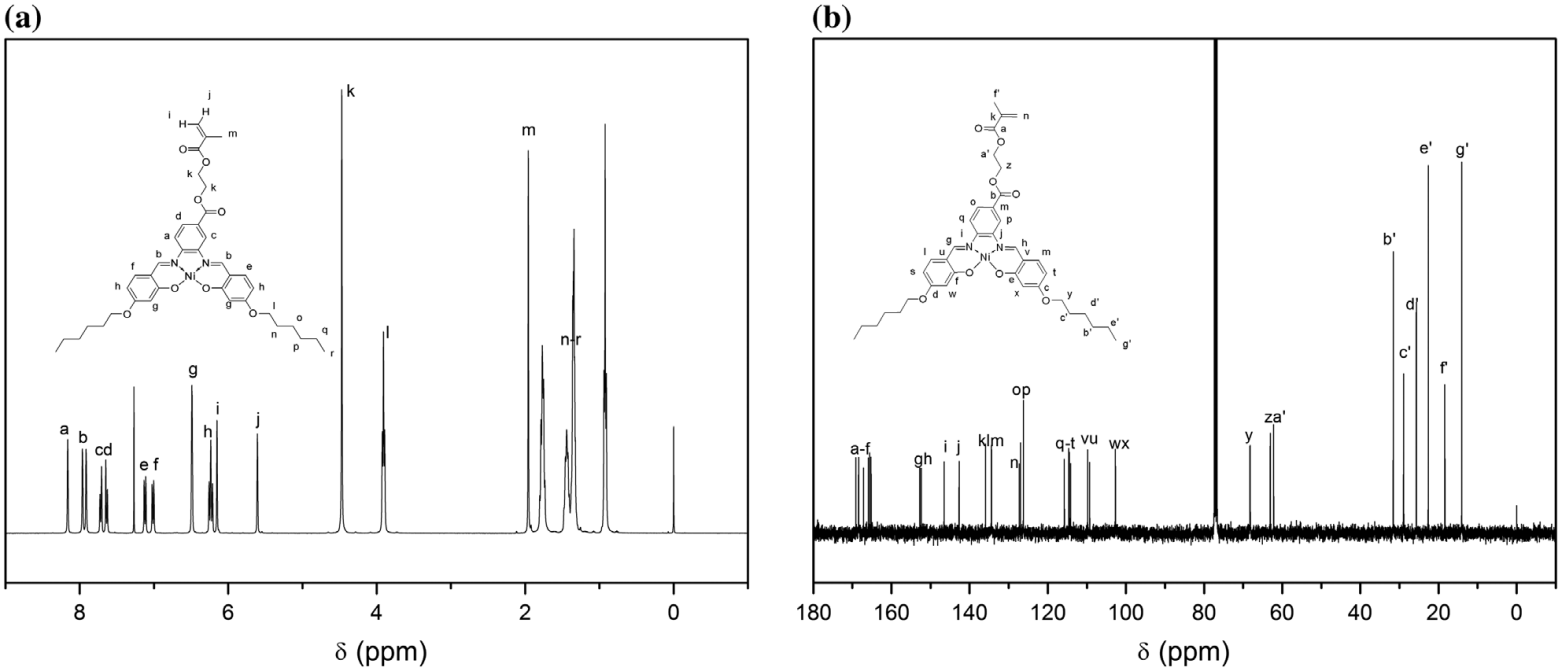
^1^H-NMR (a) and ^13^C NMR (b) spectra of HSNi.

As shown in Figure 1(a), all protons were clearly assigned, consistent with the monomer structure. Signals appeared at the peaks of 6.15 and 5.61 ppm were corresponded to vinyl protons from methacrylate double bonds while the peaks at 6.23–8.16 ppm were corresponded to the benzene rings of the salphen unit. The two peaks at 4.47 and 3.91 ppm were assigned to the ethylene protons of HSNi. Chemical shifts of the multipeaks at 0.92–1.77 ppm were assigned to the substituted alkyl group. The ^13^C NMR spectrum of HSNi (Figure 1(b)) was consistent with the monomer structure, further demonstrating the successful synthesis of HSNi monomer.

### 3.2. Polymer synthesis

**Figure 2.**
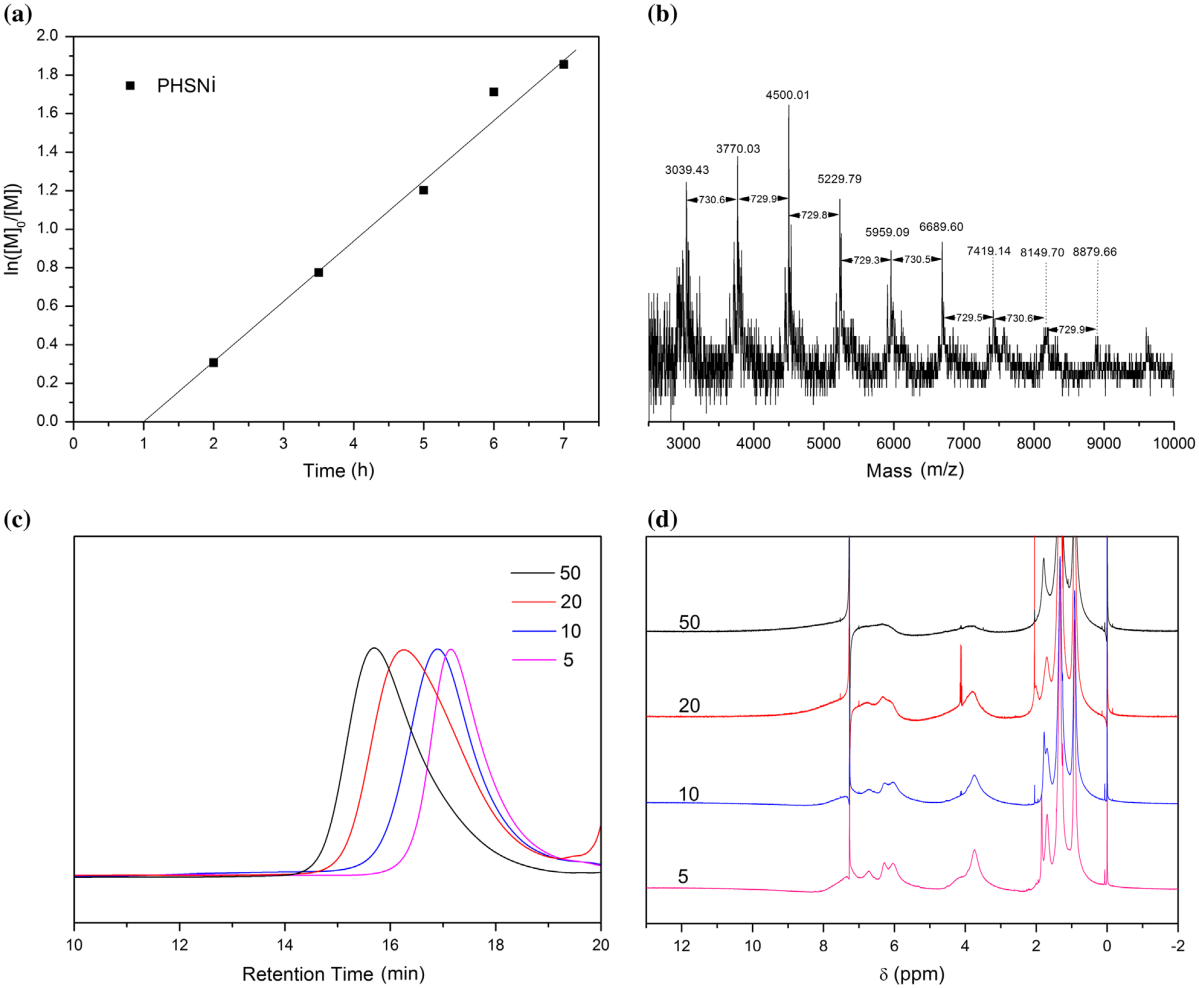
(a) Semilogarithmic plot of polymerization of HSNi by RAFT. (b) MALDI-ToF spectrum of PHSNi. GPC curves (c) and ^1^H-NMR spectrum (d) of PHSNi with different DPs.

**Figure 3.**
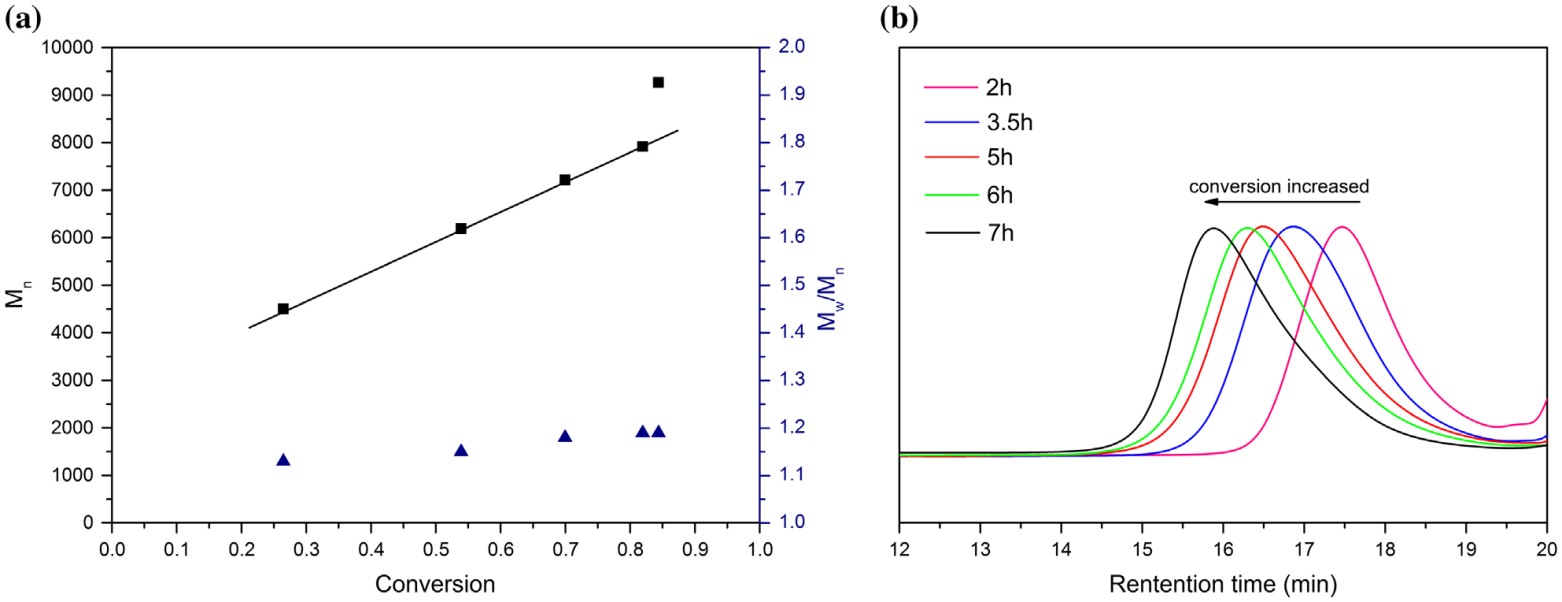
(a) Plots of PHSNi molecular weight (M_n_, GPC), PDI (GPC) vs. monomer conversion (^1^H-NMR). (b) GPC curves of PHSNi with different conversion.

RAFT polymerization was utilized to synthesize side-chain Salphen-nickel-containing homopolymer from monomer HSNi. According to a RAFT polymerization protocol, CDB was used as the RAFT agent and AIBN was used as the initiator in THF at 65 °C. A degree of polymerization (DP) of 50 was targeted. As a result, a nickel-containing polymer PHSNi as a maroon powder with the molecular weight of 13,000 g/mol and Polydispersity Index (PDI) of 1.22 was obtained. Demonstrated by the linear relationship between ln([M]_0_/[M]) ([M]_0_ and [M] are the monomer concentrations at the beginning and at a given time, respectively) and reaction time (Figure 2(a)), the process was a controlled/living polymerization after an hour of induction period. As shown in Figure 3, the molecular weight increased linearly with conversion. Meanwhile, the PDIs were below 1.16 during the polymerization, which also indicated the well-controlled nature of the polymerization.

PHSNi has a high solubility in common organic solvents, such as benzene, chloroform, THF and methylene chloride, because the alkyl substituents on salphen could promote the good solvent-polymer chain segment interactions and decrease the interchain interactions.

Similarly, a series of well-defined PHSNis with variant DPs were also successfully prepared. As shown in Figure 2(c), all the GPC curves were symmetrical single peaks. GPC curves shifted toward shorter retention time with increased DPs, indicating the increased molecular weights of the obtained polymers. Narrow PDIs (1.19−1.23) were indicated the well-controlled molecular weight distribution of these polymers. The aromatic hydrogen NMR peaks around 7–8 ppm (Figure 2(d)) slightly shifted toward upfield and broadened with increased DPs, demonstrating the gradually increased molecular weights and the enhanced aggregation behavior of the pendant Salphen-Ni.[39]

As listed in the MALDI-ToF spectrum (Figure 2(b)), the intervals of adjacent peaks were almost constant and equal to the molar mass of a single HSNi methyl acrylate repeat unit (729.5 g·mol^−1^), which indicated the high stability of the monomer during the polymerization process. The observed mass agreed well with the calculated molecular weight of PHSNi (729.5 n) bearing cumyl groups (119 g mol^−1^) in the end, where n is the degree of polymerization. Thiocarbonylthio moiety was decomposed in the ionization process as presented in other reports.[40]

### 3.3. Electrochemical properties of polymer

**Figure 4.**
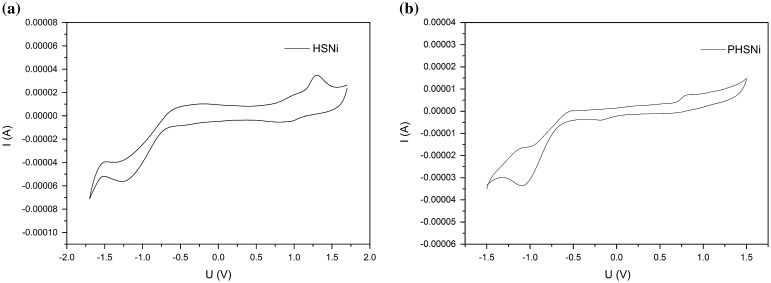
Cyclic voltammetry curves in degassed CH_2_Cl_2_ with TBAPF_6_ as supporting electrolyte, scan rate = 100 mV/s: (a) HSNi, (b) PHSNi.

In order to better understand the electrochemical properties of HSNi, a model molecule SalophenNi was prepared (Figure S8) and similar redox behavior was found. Studies on the electrochemistry of HSNi and PHSNi showed that these compounds were capable of both reduction and oxidation. As shown in Figure 4, monomer HSNi and polymer PHSNi displayed an reversible reduction peak at −1.2 and −1.0 V respectively vs. the saturated calomel electrode (SCE), due to the reduction of salphen-Ni to [salphen-Ni]^−^.[38] HSNi showed a reversible oxidation peak at 1.25 V vs. the SCE, which can be attributed to the oxidation of salphen-Ni to [salphen-Ni]^+^, an nickel–radical species.[41] Compared with HSNi, the curve PHSNi was irreversible in oxidation, The irreversibility might because of the solubility change of HSNi under redox process.[22]

### 3.4. Thermal properties of polymer

**Figure 5.**
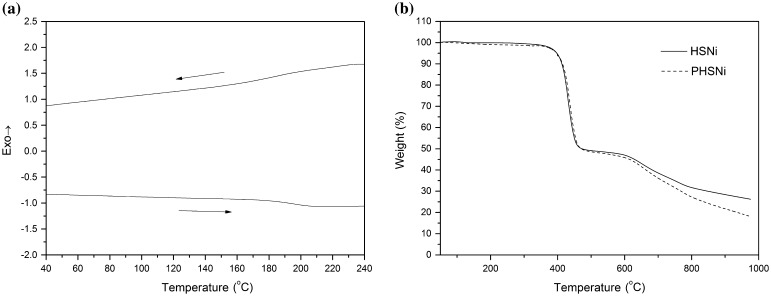
DSC (a) and TGA (b) curves of PHSNi.

The thermal property, which is an important parameter related to polymer’s stability and aging resistant, was carefully studied. As shown in the thermogravimetric analysis (TGA) (Figure 5(b)), both monomer and polymer exhibited two obvious weight loss. At the temperature range of 410–450 °C, the weight loss percentage was around 50 wt%, corresponding to the degradation of the subsitituted alkyl chain and polymer skeleton. Above 600 °C, the decomposition of the benzene rings caused the second weight loss. Compared to the monomer, the polymer degraded faster when the temperature was higher than 600 °C, which might be due to the different microstructures effected by polymerization. DSC measurement (Figure 5(a)) showed that PHSNi displays a glass transition temperature at 176 °C, which is higher than that of most metallopolymers.[19,20,22]

## 4. Conclusions

In summary, a novel methacrylate-containing nickel complex was first successfully synthesized. Controlled RAFT polymerizations were further carried out to prepare a series of side-chain nickel containing polymers with different molecular weight and narrow PDI. Electrochemical and thermal properties were also investigated for this new metallopolymer, and more fascinating properties were waiting to be discovered. Consequently, this study provided a novel and convenient strategy to prepare metallopolymers with controllable molecular weight. Just as metallocene polymers, this novel side-chain nickel containing polymer will flourish gradually in the field of metallopolymers.

## Supplemental data

The supplemental data for this article is available online at http://dx.doi.org/10.1080/15685551.2016.1257378.

## Funding

This work was supported by National Science Foundation of China [grant number 21174129].

## Disclosure statement

No potential conflict of interest was reported by the authors.

## Supplementary Material

TDMP_1257378_Supplemental_Material.pdf
