# Supplementary material for: Facile preparation of a novel nickel-containing metallopolymer via RAFT polymerization
Source: Des Monomers Polym. 2016 Nov 21;20(1):300–7. doi: 10.1080/15685551.2016.1257378 (PMC5812193; doi:10.1080/15685551.2016.1257378)
Supplement: TDMP_1257378_Supplemental_Material.pdf [file TDMP_A_1257378_SM2443.pdf]

Supporting Information

Facile Preparation of a Novel Nickel-Containing Metallopolymer via RAFT Polymerization

Rong Ren, Yanhua Wang, Dizheng Liu, Weilin Sun*

Supporting Information

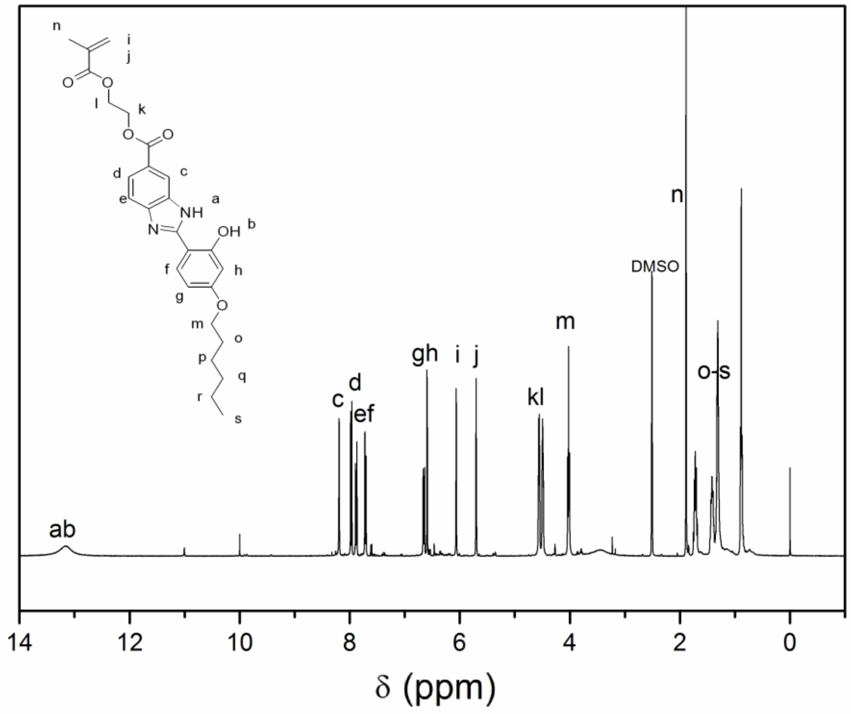

Figure S1. ^{1}H NMR spectrum of benzimidazole derivatives.

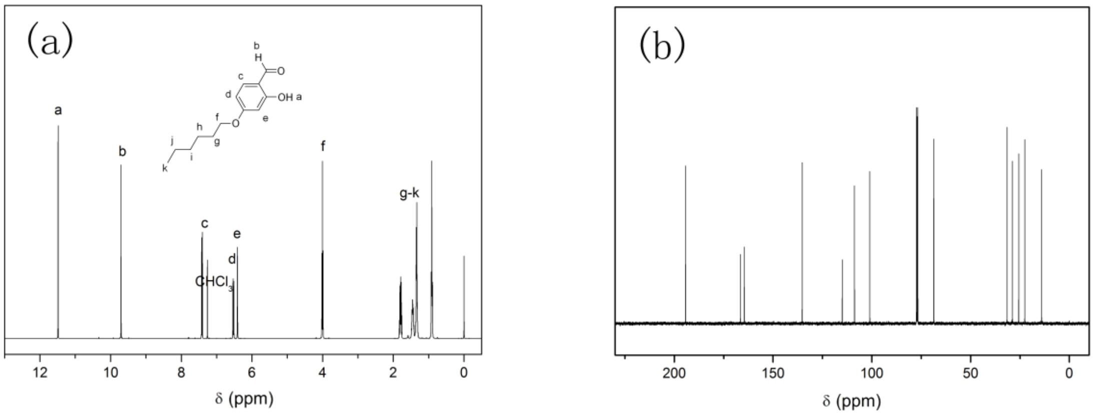

Figure S2. ^{1}H NMR (a) and ^{13}C NMR (b) spectra of 4-Hexyloxysalicylaldehyde.

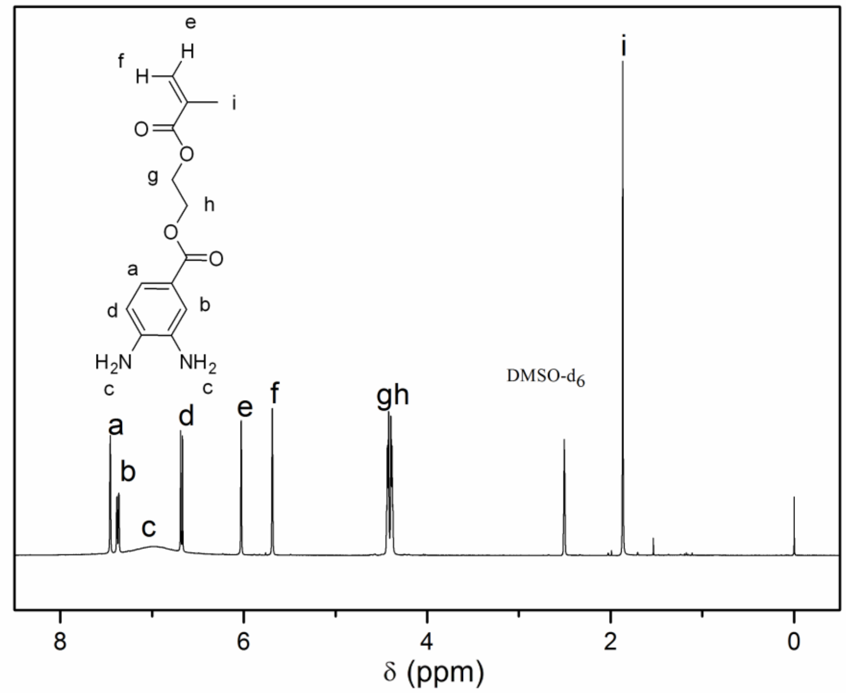

Figure S3. ^{1}H NMR spectrum of HEMA-DBBA.

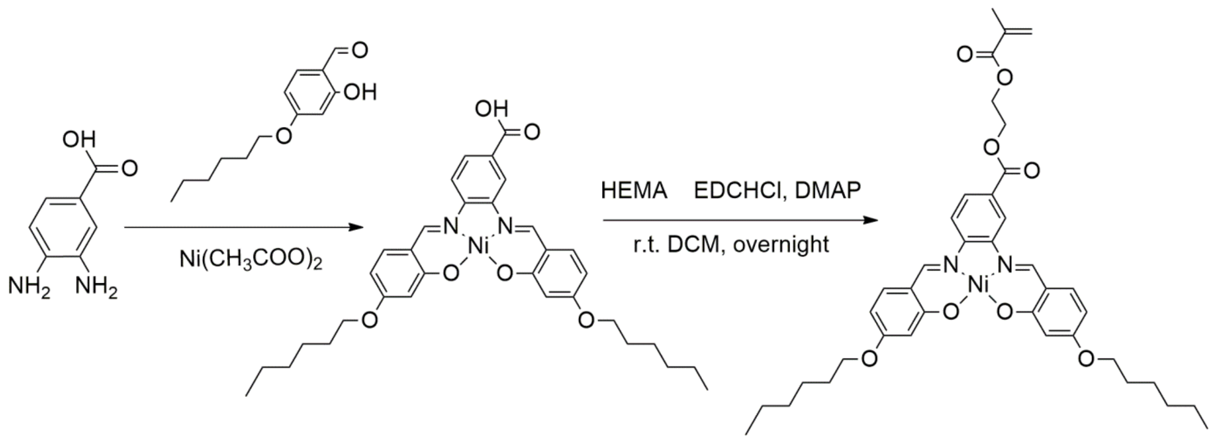

Scheme S1. Synthetic Route to HEMA-Salphen-Ni.

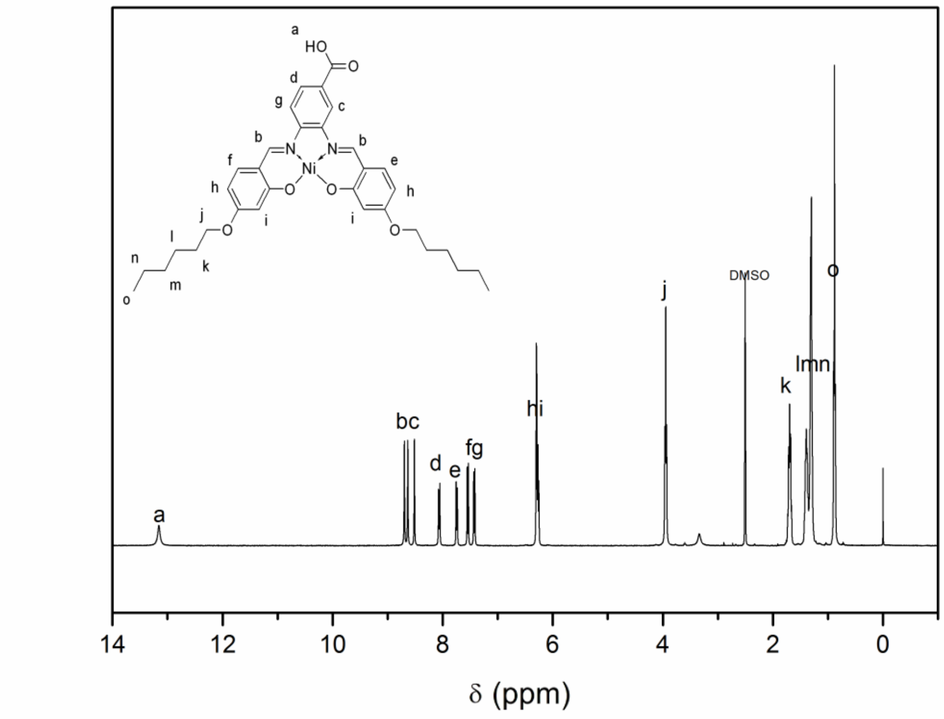

Figure S4. ^{1}H NMR spectrum of SalphenNi-COOH.

Attempted synthesis of HEMA-Salphen-Cu (HSCu), HEMA-Salphen-Zn (HSZn) and their polymers

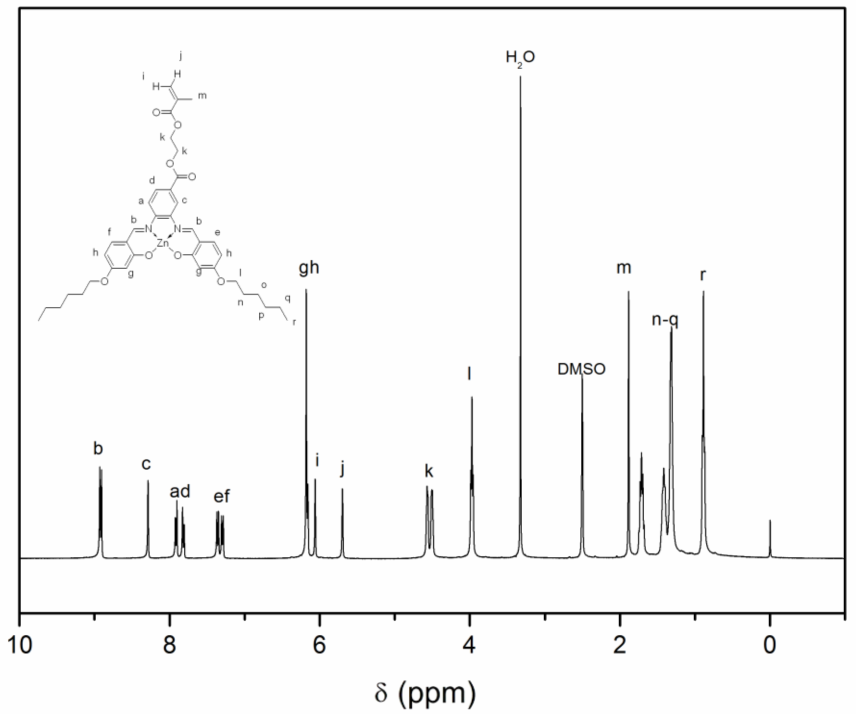

Figure S5. ^{1}H NMR spectrum of HSZn.

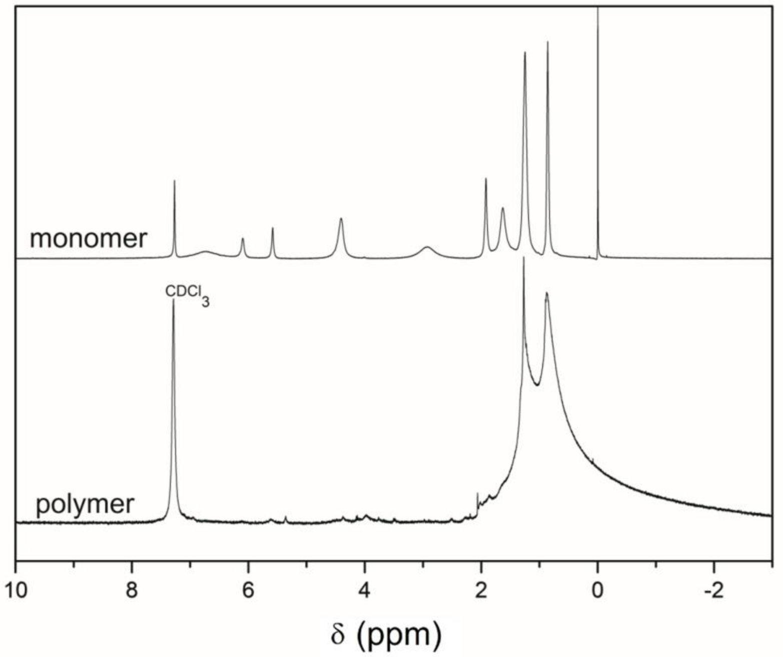

Figure S6. ^{1}H NMR spectra of HSCu and PHSCu.

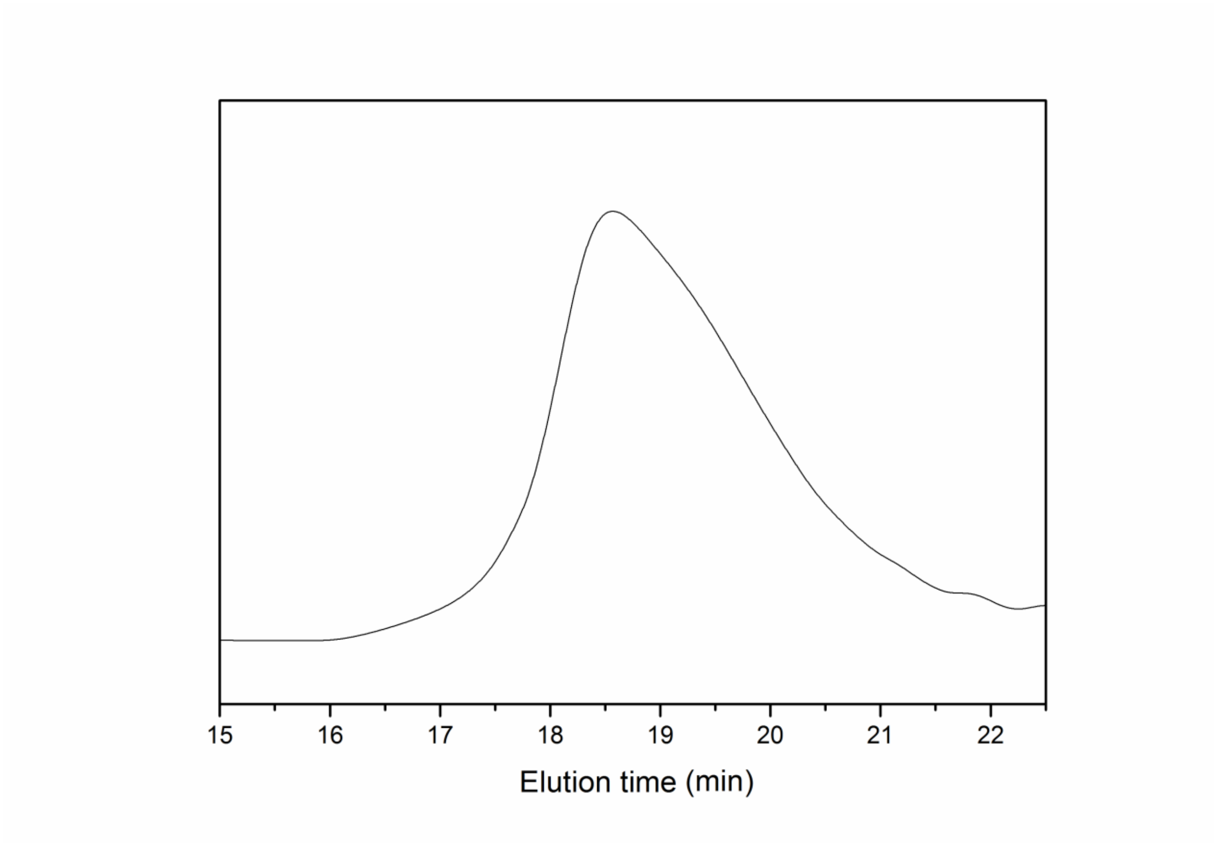

Figure S7. GPC curve of PHSCu with Mn=2380, Mw/Mn=1.39.

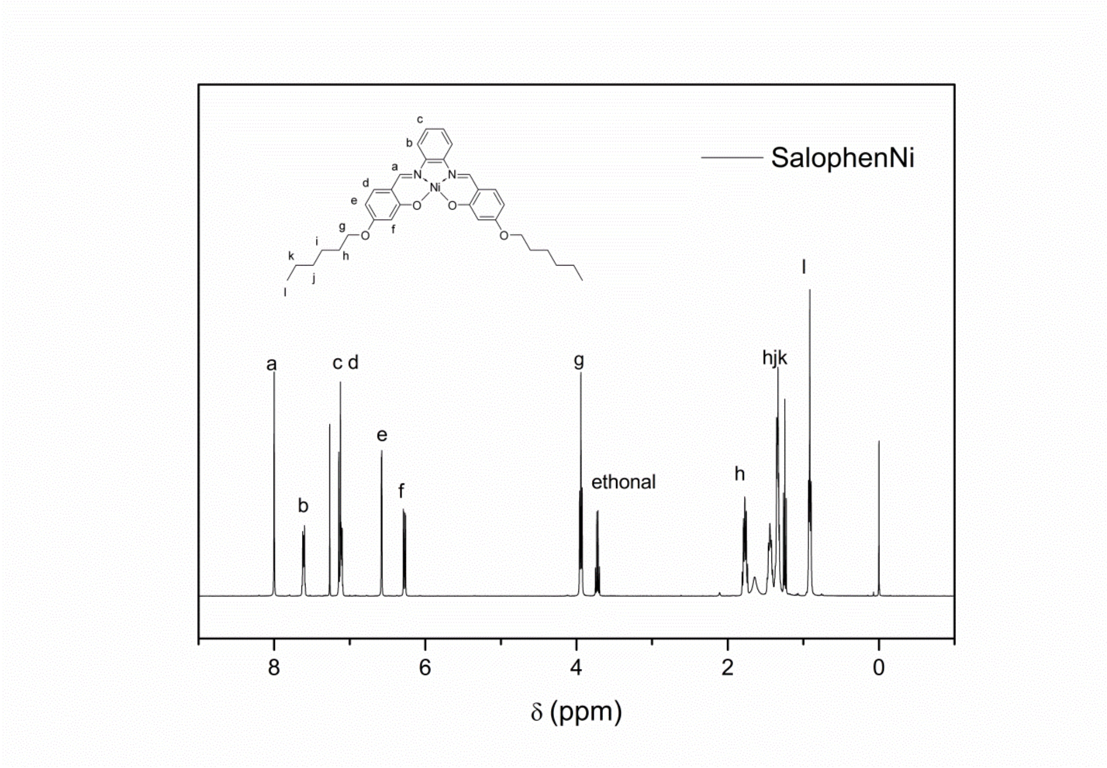

Figure S8. Structure and ^{1}H NMR spectrum of SalophenNi.

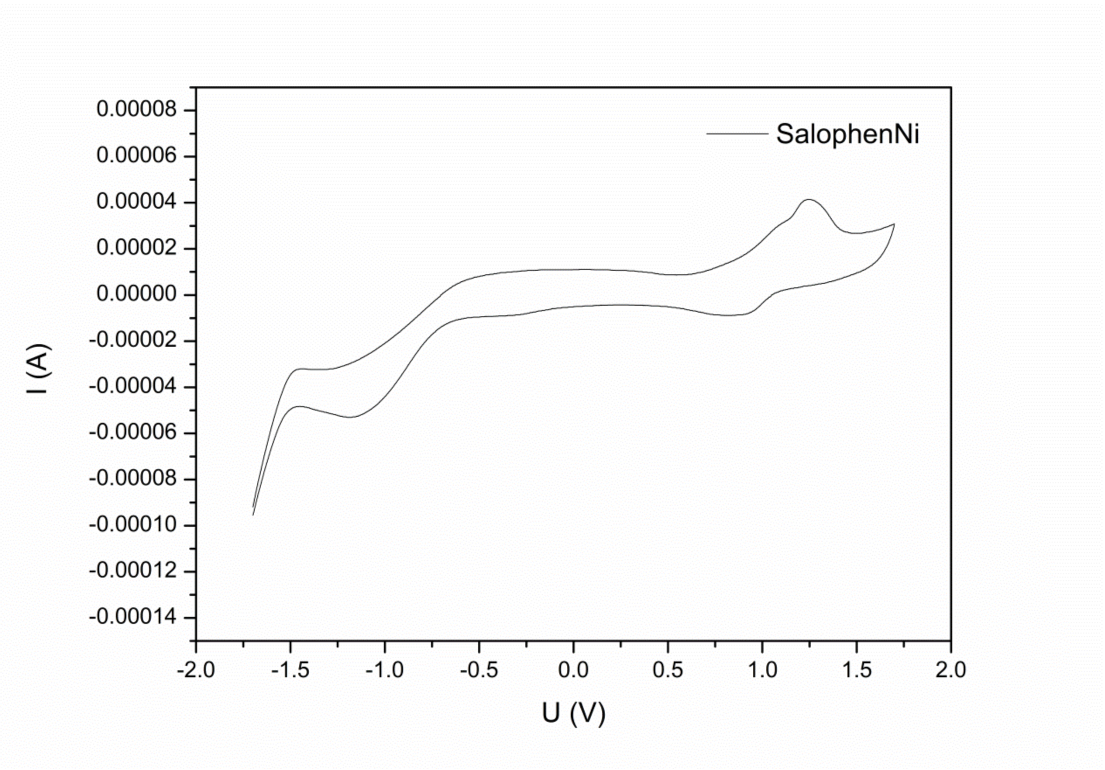

Figure S9. Cyclic voltammetry curve of SalophenNi in degassed CH_2Cl_2 with $TBAPF_6$ as supporting electrolyte, scan rate = 100 mV/s.

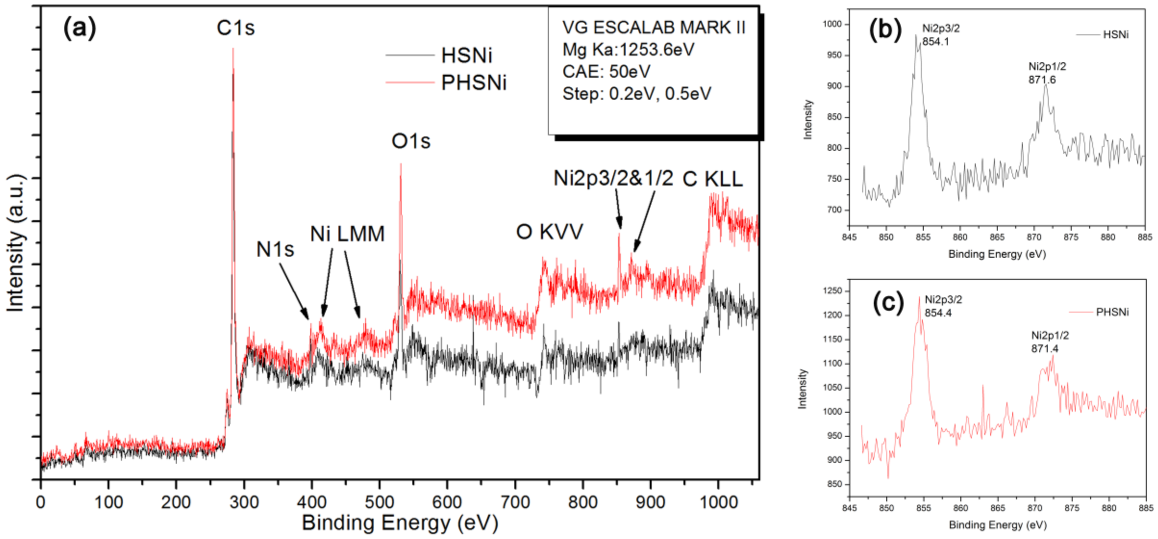

Figure S10. XPS spectra of HSNi and PHSNi (a); expanded view of HSNi (b) and PHSNi (c) in the region from 845 to 885 eV.
